# Isolation of an “Early” Transit Amplifying Keratinocyte Population in Human Epidermis: A Role for the Low Affinity Neurotrophin Receptor CD271

**DOI:** 10.1093/stmcls/sxac060

**Published:** 2022-08-29

**Authors:** Roberta Lotti, Elisabetta Palazzo, Marika Quadri, Marc Dumas, Sylvianne Schnebert, Diego Biondini, Maria Anastasia Bianchini, Carine Nizard, Carlo Pincelli, Alessandra Marconi

**Affiliations:** DermoLab, Department of Surgical, Medical, Dental and Morphological Sciences, University of Modena and Reggio Emilia, Modena, Italy; DermoLab, Department of Surgical, Medical, Dental and Morphological Sciences, University of Modena and Reggio Emilia, Modena, Italy; DermoLab, Department of Surgical, Medical, Dental and Morphological Sciences, University of Modena and Reggio Emilia, Modena, Italy; LVMH Recherche, Life Sciences Department, Saint Jean de Braye, France; LVMH Recherche, Life Sciences Department, Saint Jean de Braye, France; Pediatric Surgery Unit, Department of Pediatric Surgery, University of Modena and Reggio Emilia, Modena, Italy; Pediatric Surgery Unit, Department of Pediatric Surgery, University of Modena and Reggio Emilia, Modena, Italy; LVMH Recherche, Life Sciences Department, Saint Jean de Braye, France; DermoLab, Department of Surgical, Medical, Dental and Morphological Sciences, University of Modena and Reggio Emilia, Modena, Italy; DermoLab, Department of Surgical, Medical, Dental and Morphological Sciences, University of Modena and Reggio Emilia, Modena, Italy

**Keywords:** skin, epidermal stem cells, early TA cells, CD271, epidermal differentiation, epidermal regeneration, senescence

## Abstract

In the interfollicular epidermis (IFE), stem cells (KSC) generate transit amplifying (TA) cells that, after symmetric divisions, produce differentiating daughters. Here, we isolated and characterized the highly proliferative interfollicular epidermal basal cell population “early” TA (ETA) cells, based on their capacity to adhere to type IV collagen. Proliferation and colony-forming efficiency in ETA cells are lower than in KSC but higher than in “late” TA (LTA). Stemness, proliferation, and differentiation markers confirmed that ETA cells display a unique phenotype. Skin reconstructs derived from ETA cells present different features (epidermal thickness, Ki67, and Survivin expression), as compared to skin equivalents generated from either KSC or LTA cells. The low-affinity neurotrophin receptor CD271, which regulates the KSC to TA cell transition in the human epidermis through an on/off switch control mechanism, is predominantly expressed in ETA cells. Skin equivalents generated from siRNA CD271 ETA cells display a more proliferative and less differentiated phenotype, as compared to mock-derived reconstructs. Consistently, CD271 overexpression in LTA cells generates a more proliferative skin equivalent than mock LTA cells. Finally, the CD271 level declines with cellular senescence, while it induces a delay in p16INK4 expression. We conclude that ETA cells represent the first KSC progenitor with exclusive features. CD271 identifies and modulates ETA cells, thus participating in the early differentiation and regenerative capacity of the human epidermis.

## Graphical Abstract

**Figure:**
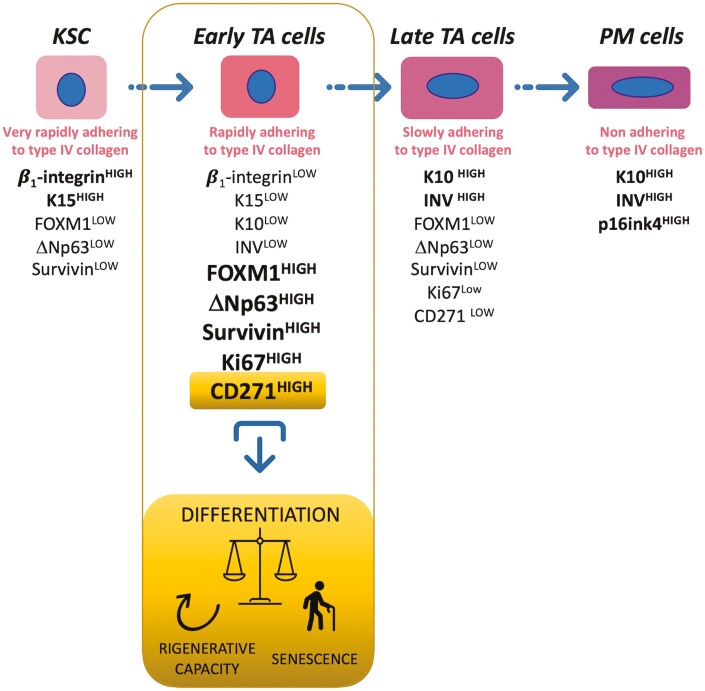

**Significance Statement**

The early events involved in the differentiation process of the human epidermis are not fully understood because of the difficulty in discriminating keratinocyte stem cells (KSC) from their progenitors. In this study, we identified an “early” transit amplifying cell (ETA) population with unique features, as compared to both KSC and TA cells. In vivo, while KSC are quiescent, ETA are active and proliferating cells. By contrast, freshly isolated KSC show a higher clonogenic potential in culture. ETA cell-derived 3D reconstructs display skin regenerative capacity inferior to KSC, but greater than TA cells. CD271 is exclusively expressed in ETA cells and modulates their functions. CD271 expressing ETA cells are an essential part of the niche and participate in the early differentiation process of the human keratinocytes.

## Introduction

Human epidermal homeostasis and maintenance depend on adult stem cells that have an unlimited self-renewal ability and contribute to skin regeneration.^1^ The classical model consists of slow-cycling stem cells that divide asymmetrically to give rise to one stem and one transit amplifying (TA) cell. TA cells, after few rounds of division, become differentiated cells.^2,3^ On the other hand, lineage tracing data show that the basal layer of the epidermis contains a single equipotent population of progenitor cells that contribute to long-term tissue maintenance by randomly balancing proliferation and differentiation.^4^ More recently, Mascrè and co-workers reestablished the keratinocyte stem (KSC)/progenitor model demonstrating, by quantitative analysis of clonal fate data and proliferation dynamics, the presence of the hierarchy comprising slow-cycling KSC and committed progenitor cells.^5^ Moreover, quiescent KSC proliferate only after TA cells are generated, indicating the latter cells as an active signal within the niche.^6^ In contrast to the quiescence state of KSC, TA cells are highly proliferative and are essential to forming new layers during epidermal stratification and in regeneration after injury.^7^ Yet, little is known about the early events occurring during the transition from KSC to more differentiated progenitors. Moreover, distinguishing KSC from TA cells remains a technical challenge, particularly because of the lack of reliable markers.

β1-integrin expression and function have been originally used to separate KSC from TA.^8^ β1-integrin signaling protects KSC from apoptosis,^9^ and blocking this receptor induces anoikis through caspase-8 activation in human keratinocytes.^10^ More specifically, we have shown that the β1B-integrin isoform is responsible for cell death in keratinocytes.^11^ β_1_-integrin signaling also maintains cell survival and controls proliferation and apoptosis of human KSC progenitors.^12^

Neurotrophins (NTs) are a family of structurally and functionally related proteins that play a critical role in the survival, differentiation, and apoptosis in the nervous system.^13^

NT functions are mediated by the low-affinity CD271 (p75NTR) and the tyrosine kinase (Trk) family of receptors.^14^ CD271 belongs to the tumor necrosis factor receptor superfamily and shares with the other members the “death domain”.^15^ Most skin cells synthesize and release NT and express NT receptors, contributing to a network of autocrine and paracrine activities.^16^ In the human epidermis, where it acts independently of Trk, CD271 is predominantly expressed in TA cells, mediates apoptosis, and differentiation.^17^ We have shown recently that CD271 also regulates the transition from KSC to TA cells in the human epidermis.^18^ When we separated KSC from TA, β1-integrin inversely correlated to CD271 silencing or overexpression.^18^ Here we report the isolation of an early TA cell subpopulation (ETA), based on the expression levels of β1-integrin. Cells were further phenotypically and functionally characterized. Finally, we present evidence that CD271 identifies ETA and plays a critical role in the early differentiation, regeneration, and senescence of the human epidermis.

## Materials and Methods

### Cell Culture

**Figure 1.**
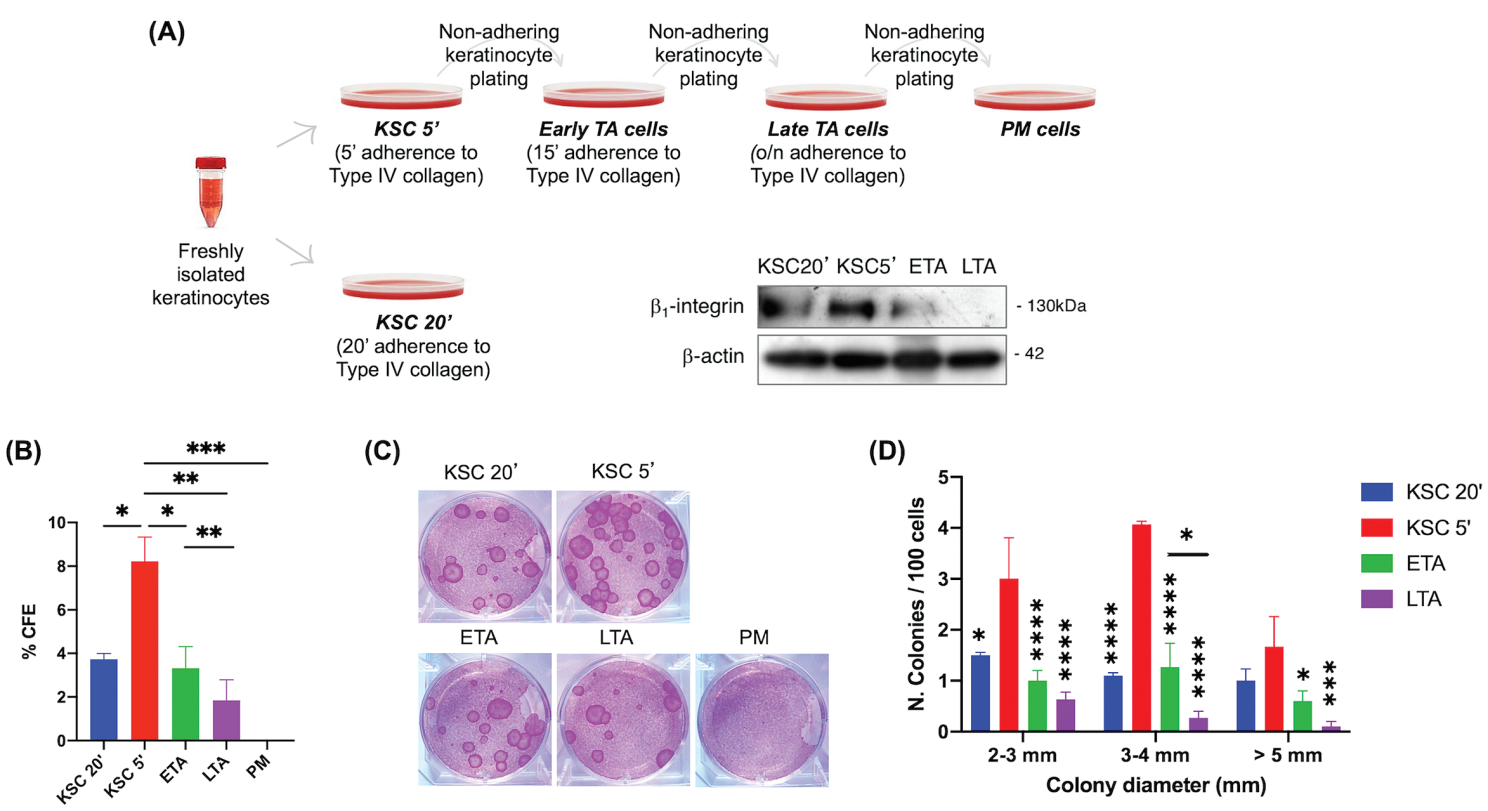
Isolation and characterization of keratinocyte subpopulations. (**A**) Keratinocytes were isolated based on their ability to adhere to type IV collagen. Four subpopulations were obtained: (1) KSC that adheres to the collagen in 5ʹ; (2) ETA cell that adheres in 15ʹ; (3) LTA cell that adheres overnight; (4) PM cell that does not adhere after 1 night. For comparison, KSC were isolated after 20ʹ. The relative β1-integrin expression of each subpopulation is shown by WB analysis. (**B**) Freshly isolated KSC 20ʹ, KSC 5ʹ, ETA, LTA, and PM cells were plated on the 3T3 feeder layer and CFE (**C**) and colony areas (**D**) were determined as described in Material & Methods section. Data are represented as mean ± SEM. Ordinary one-way ANOVA followed by Tukey’s multiple comparisons are represented. Comparisons with KSC5ʹ, unless differently indicated, are shown. ns: *P* > .05; * .01 < *P* < .05; ** *P* < .01; ***P < .001; ****P < .001.

Human keratinocytes were obtained from neonatal foreskin or from normal human adult epidermal keratinocytes (NHEK) isolated from female skin. All samples were collected with written informed consent of patients, according to the Declaration of Helsinki after approval of the Modena Medical Ethical Committee (Prot. 184/10). Fresh keratinocytes were separated in subpopulations on the basis of their ability to adhere to type IV collagen 100 μg/mL (Sigma, St. Louis, MO, USA), as described in Fig. 1A modifying Tiberio et al.^9^ Keratinocytes were analyzed immediately after the separation or cultured in Keratinocyte Serum-Free Medium Epilife (MEPI500CA, Life Technologies, Carlsbad, CA, USA) supplemented with HKGS (Life Technologies). For the replicative senescence experiment, NHEK were cultivated for several passages (P), until passage 9 (P9).

### MTT Assay

Freshly isolated keratinocyte subpopulations were seeded in a 96-well tissue culture plate (5000 cells/well), and MTT (3-(4,5-dimethylthiazol-2-yl)-2,5-diphenyltetrazolium bromide, Sigma) assay was performed up to 196 h after plating. The results are normalized on 24 h values of MTT positive cells.

### Colony Forming Efficiency (CFE) Assay

Freshly isolated keratinocyte subpopulations were plated at a density of 100 cells/dish in a 6 well-plate on mitomycin C-treated 3T3 cells (2.4 × 104/cm^2^) and cultivated in DMEM and Ham’s F12 media.^18^ Fourteen days later, dishes were fixed and colored with 4% formaldehyde/1% Rhodamine B. Colonies that contained more than 10 cells were counted, and CFE was calculated. The colony number was expressed as a percentage of the number cells of plated. Colonies were further analyzed according to their diameter.

### Long-Term Assay

Freshly isolated keratinocyte subpopulations were plated at the density of 1000 cells/cm^2^ in a keratinocyte serum-free medium. At the 80% confluence, cells were serially passaged at the same density (1000 cells/cm^2^) until the growth capacity of the cells was exhausted. The total cell number of cells obtained at each passage was calculated. In addition, population doubling capacity was calculated as follows


$$\begin{array}{l} PD=3.322_{*log}10\left(\frac{N\;cells\;Pn}{N\;cells\;P1}\right) \end{array}$$

### Skin Reconstructs

Skin reconstructs were obtained by seeding freshly isolated keratinocytes on dermal equivalents generated by fibroblasts-induced type I collagen contraction, as described in Truzzi et al.^18^ After 6 days or 12 days, skin reconstructs were fixed with 4% formalin for 2 hours at room temperature, dehydrated, and embedded in paraffin.

### siRNA Transfection of ETA Cells

ETA keratinocytes were grown at sub confluency. Cells were then transfected with 50 nM CD271 or scrambled siRNA (Dharmacon Inc, Lafayette, CO, USA), as previously described.^18^ After 48 h cells were used for Western blotting (WB) or for skin equivalent setting up.

### Infection of ETA or LTA Cells

ETA or LTA cells were transduced by infection with viral supernatant generated by CD271-LNSN packaging cells or by LNSN packaging cells (kindly provided from F. Mavilio) in the medium for keratinocytes in the presence of polybrene (8 μg/mL).^17^ Forty-eight hours after infection, cells were lysed for WB analysis or used for skin equivalent setting up.

### SA-β-Gal Staining

We used the SA-β-Gal staining kit (Sigma–Aldrich) to determine the replicative senescence according to Itahana et al.^19^ This β-galactosidase activity at pH 6.0 is present only in senescent cells. In presence of β-galactosidase, the X-Gal substrate (5-bromo-4-chloro-3-indolyl-β-D-galactopyranoside) is hydrolyzed and changes its color to blue due to the formation of 5-bromo-4-chloro-3-indol. The cells were incubated in 1 × fixation buffer for 6 min at room temperature, washed twice with 1 × PBS, and incubated overnight with staining solution at 37 °C without CO_2_. Results were presented as a percentage of blue positive cells.

### Western Blotting Analysis (WB)

Freshly isolated keratinocyte populations were harvested for CD271 in lysis buffer pH 7.5 (150 mM NaCl, 15 mMMgCl, 1 mM EGTA, 50 mM Hepes, 10% Glycerol, 1% Triton), or in RIPA buffer. WB was performed as previously described.^9^ Membranes were first incubated in blocking buffer and then overnight at 4 °C with primary antibodies: mouse anti-human CD271, clone ME20.4 (Upstate, Lake Placid, NY, USA), mouse anti-human β1 integrin, clone 12G10 (Abcam, Cambridge, UK), rabbit anti-FOXM1, clone D3F2B (Cell Signaling Tech, Danvers, MA, USA), rabbit anti-p63(ΔN) (BioLegend, San Diego, CA, USA), rabbit anti-Survivin (Boster, Pleasanton, CA, USA), rabbit anti-p16INK4 (Bethyl Laboratories Inc, Montgomery, TX, USA), rabbit anti-Ki67 (Abcam), mouse anti-human β-actin, clone AC-15 (Sigma). Then membranes were incubated with anti-mouse or anti-rabbit peroxidase-conjugated secondary antibodies (Biorad, Hercules, CA). Membranes were developed in Clarity Max ECL substrate (BioRad) and images were captured with ChemiDoc Imaging System (Biorad). The band intensity was quantitatively determined using Fiji-ImageJ software (Wayne Rasband, National Institute of Mental Health, Bethesda, MD, USA), and protein levels’ intensity was normalized to β-actin expression.

### Automated Capillary Electrophoresis Western Analysis

Normal Human Epidermal Keratinocytes were lysed in whole cell extraction buffer (25 mM Tris HCL buffer pH 7.5; 0.25 M saccharose 0.2 mM MgSO4; 20 mM EDTA; 0.4 % Triton X-100; 2 mM DTT; 5 µg/mL leupeptin; 0.4 mM PMSF). CD271 protein levels were determined by capillary electrophoresis immunoassay by following the WES user guide from ProteinSimple and after determination of primary antibody dilution. Cell extracts samples (1.92 µg of protein/lane) were mixed with a 5 × Master Mix (DTT, fluorescence labeled maker, SDS) and then heated at 70 °C for 10 min. The samples, the biotin labeled protein ladder (12 kDa, 40 kDa, 66 kDa, 116 kDa, 180 kDa, and 230 kDa), blocking reagent, CD271 Receptor antibody at a dilution of 1:100 (NBP2-19669, Novus Biologicals Europe), HRP-conjugated secondary antibody, luminol S/peroxide, and separation and stacking matrices were also dispensed to designated wells plate. After plate loading, the electrophoresis and immunodetection steps took place in the capillary system (ProteinSimple WES, Santa Clare, CA, USA) and were fully automated with instrument default settings. Peak areas were determined using Compass software (Protein Simple) and normalized to β-actin (loading control) (NB600-532, Novus Biologicals Europe).

### Immunofluorescence (IF)

Freshly isolated keratinocyte subpopulations were fixed in 4% buffered PFA and cytospun onto glass slides. Cells were permeabilized with Triton × 100 0.1%, stained for K10 (clone EP1607Y, Epitomics, Burlingame, CA, USA), K15 (EPR1614Y, Epitomics), and Involucrin (clone I9018, Sigma) and then labeled with Alexa Fluor secondary antibodies, Alexa Fluor 546 and 488-conjugated goat IgGs (Invitrogen, Waltham, MA, USA). Then slides were stained with 1 μg/mL Dapi (Sigma). Immunofluorescence on skin equivalent slides was performed as in IHC methods except for the different primary antibodies: K10, K15, and Survivin (Novus Biologicals, Littleton, CO, USA) and the secondary antibodies Alexa Fluor 546 and 488-conjugated goat IgGs (Invitrogen). Micrographs were taken on a Confocal Scanning Laser Microscopy (Leica TCS4D) (Leica, Exton, PA, USA). Quantification of immunofluorescence staining was performed by analyzing 6 representative fields for each staining sample, using ImageJ software. Scoring was made by means of positive cell counting.

### Immunohistochemistry (IHC)

Paraffin-embedded biopsies (4 µm) of normal human skin and skin reconstructs were stained for protein analysis by immunohistochemistry as previously described.^20^ Sections were stained with H&E or stained for Ki67 (clone MIB-1, Dako, Glostrup, Denmark) with 3,3ʹdiaminobenzidine (DAB) as cromogen, according to ultraView Universal DAB detection kit instructions (Ventana, Oro Valley, AZ, USA). Images of the immunohistochemistry staining were obtained by a D-Sight slide scanner (Menarini Diagnostics, Firenze, Italy). The epidermal thickness was quantitatively determined using D-Sight and ImageJ software. The number of suprabasal and basal positive nuclei for Ki67 was counted and expressed as a number of positive nuclei/basal cells. This was performed on at least 3 different sections and the results were reported as a mean.

### RNA Extraction, Reverse Transcription, and Real-Time qPCR

Total RNA from cultured cells was extracted using a NucleoSpin RNA kit (740709; Macherey Nagel, Düren, Germany). Reverse transcription was performed on 500 ng of total RNA using a High Capacity cDNA Reverse Transcription Kit (4368813, Applied Biosystems). Real-time qPCR was achieved with the 7900HT Fast Real-Time PCR System with 96 Well Block Module (Applied Biosystems). The mRNA expression of 3 target genes was analyzed using predesigned and optimized inventoried assays (Applied Biosystems) according to the manufacturer’s instructions. The following assays were used: (Hs00606991_m1) Ki67 marker of proliferation; (Hs00923894_m1) P16 and (Hs01121172_m1) P21 markers of cyclin dependent kinase inhibitor; and to normalize gene expression, β-2-microglobulin (B2M) (Hs99999907_m1) was used as a housekeeping gene.

### Cell Cycle Analysis

Freshly isolated keratinocytes were resuspended in hypotonic fluorochrome solution containing 50 µg/mL propidium iodide, 0.1% sodium citrate, and 0.5% Tryton X-100 (Sigma Aldrich, Milano, Italy). After 15 min, cells were analyzed using Attune Nxt flow cytometer (Invitrogen).

### Statistical Analysis

Results are presented as means ± SEM, obtained from at least 3 different experiments. Prism Software (Graph Pad Software V9.0) was used to perform statistical analysis. A two-tailed unpaired Student’s *t*-test was used for statistical comparisons between 2 groups, while ordinary one-way ANOVA followed by Tukey’s multiple comparisons was used for more than 2 groups, as indicated in figure legends. A value of *P* < .05 or less was assumed to indicate a statistically significant difference in the compared parameters.

## Results

### Isolation and Characterization of Early TA (ETA) Cells from Human Keratinocytes

Human interfollicular stem cells (IFSC) were first identified by isolation from the skin and by culturing them in vitro. Three clonal types from single keratinocyte were characterized for their different multiplication capacity, namely holoclones with the highest proliferation rate, meroclones characterized by a lower growth potential, and paraclones with short replicative lifespan.^21^Jones and Watt first reported high expression of β1-integrin as a marker for human IFSC. This method is based on the different capacities of keratinocytes to adhere to the β1-integrin ligand type IV collagen and allows the isolation of KSC and TA cells.^8^ The method has been widely used and improved over the years, by enriching subpopulations with different genes.^22^To better identify keratinocyte subpopulations, we have modified this method by reducing the time of adherence to type IV collagen and characterizing the cells (Fig. 1A). Keratinocytes adhering for 5 min (KSC 5ʹ), express higher amounts of β1-integrin (Fig. 1A) and display a superior colony forming efficiency (CFE), as compared to the cells adhering in 20 min (KSC 20ʹ) (Fig. 1A, 1B, 1C). Moreover, colonies are strikingly more numerous in the fast-adhering cells (KSC 5ʹ), with the number of large colonies being higher than in keratinocytes adhering in 20 min (KSC 20ʹ) (Fig. 1B, 1C). Fig. 1D shows the highest number of colonies in all the dimensional ranges analyzed, in particular 3–4 mm diameter, in rapidly adhering cells (KSC 5ʹ). Our approach also allows the isolation of 2 different TA populations, the early TA (ETA) and the late TA (LTA) cells, as shown by β1-integrin levels, CFE and the dimension of colonies. In detail, β1-integrin is expressed in ETA but not in LTA cells, while CFE in ETA is statistically higher than in LTA cells that are isolated after overnight adhesion (Fig. 1B). Consistently, both small and large colonies are more numerous in ETA than in LTA cells (Fig. 1D).

**Figure 2.**
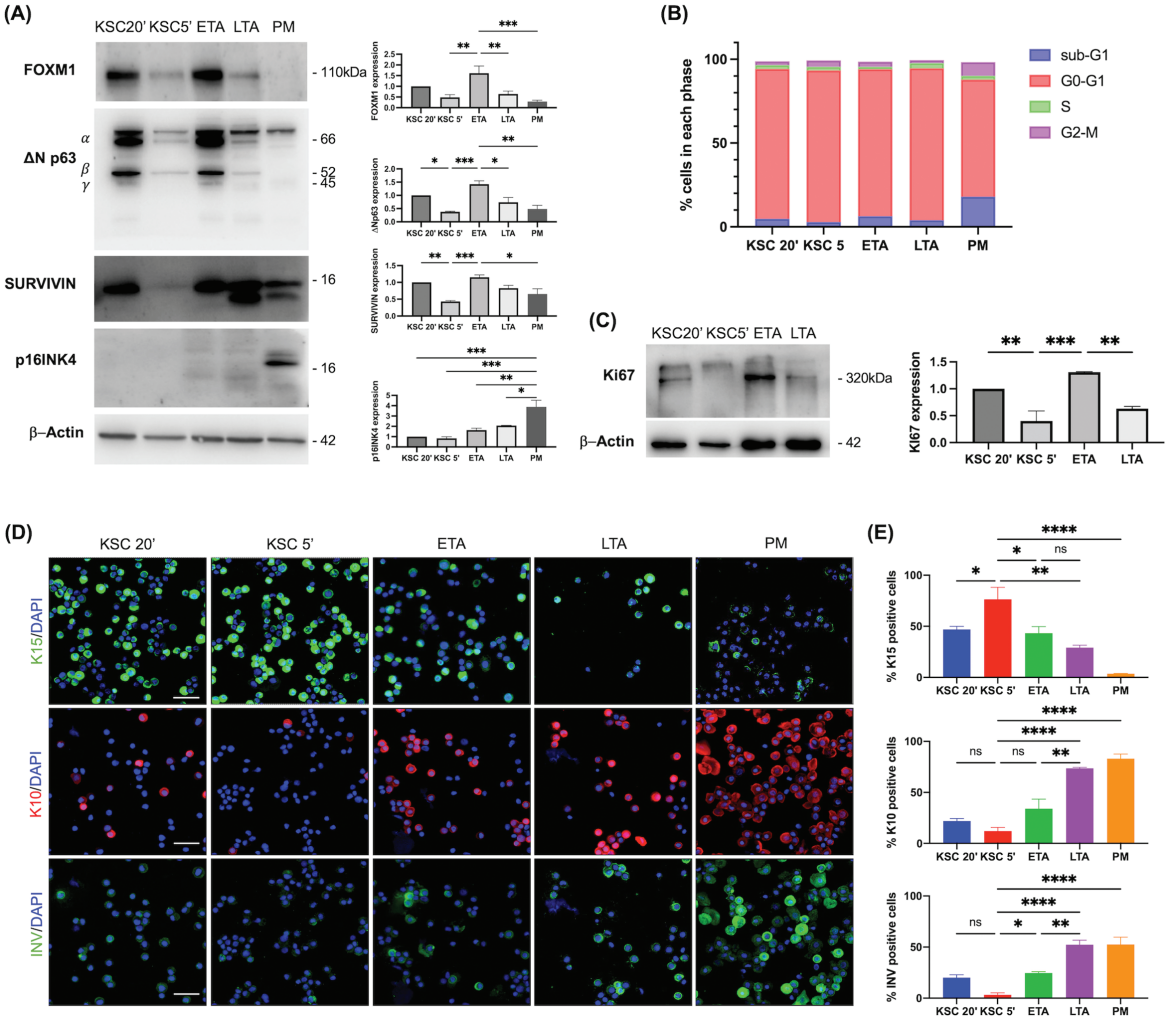
Molecular characterization of keratinocyte subpopulations. (**A**) Keratinocytes were isolated based on their ability to adhere to type IV collagen. Subpopulations were obtained: KSC20ʹ, KSC5ʹ, ETA, LTA, and PM. Cells were lysed immediately after the subpopulation isolation and the expression of FOXM1, deltaNp63 (ΔNp63), Survivin, p16INK4 were analyzed by WB. Actin was used as an internal control. Representative pictures of at least 3 different population isolations are shown. Densitometric analysis per each protein was shown (mean ± SEM, *n* = 3), evaluated by Fiji-ImageJ software. (**B**) Freshly isolated KSC 20ʹ, KSC 5ʹ, ETA, LTA, and PM cells were stained with propidium iodide (PI) solution and analyzed by flow cytometry. (**C**) Freshly isolated KSC 20ʹ, KSC 5ʹ, ETA, and LTA were lysed and Ki67 was analyzed by WB. Representative pictures of at least 3 different population isolations are shown. Densitometric analysis is shown (mean ± SEM, *n* = 3). (**D**) Freshly isolated subpopulations were double stained with DAPI and anti-K15, -K10, -Involucrin antibodies, and analyzed by confocal microscopy. Bar = 100 μm. (**E**) The number of positive cells was evaluated in at least 3 different fields. Data are represented as mean ± SEM. Ordinary one-way ANOVA followed by Tukey’s multiple comparisons are represented. ns: *P* > .05; * .01 < *P* < .05; ** *P* < .01; *** *P* < .001; **** *P* < .001.

To further characterize the subpopulations, keratinocytes were freshly isolated, and a number of markers were analyzed immediately after the population’s separation. Because FOXM1 was recently shown to be a key regulator of holoclones,^23^ and DeltaNp63 (ΔNp63) is a key transcription factor regulating the proliferative potential of epidermal stem cells,^24^ we analyzed their expression in keratinocytes subpopulations (Fig. 2A). FOXM1 is predominantly expressed in keratinocytes adhering after 20 min (KSC20ʹ) and in ETA cells, while their levels are negligible in KSC5ʹʹ, adhering after 5 min, and in LTA cells. Similarly, DeltaNp63 is expressed in KSC20ʹ and in ETA cells, but nearly absent in KSC5ʹ. Survivin, which is expressed in in vitro cycling epidermal stem cells,^23,25^ is absent in KSC5ʹ and markedly expressed in KSC20ʹ and ETA cells. Finally, p16INK4, a marker of cell senescence,^26^ is only expressed in PM cells (Fig. 2A). We next demonstrated that KSC and ETA marker expression changes depending on the proliferative state. Indeed, unlike in freshly isolated keratinocytes, FOXM1, ΔNp63, and Survivin expression were similar in all of the keratinocyte subpopulations after 3 days in culture (Supplementary Fig. S1A, S1B). To evaluate the proliferative state of keratinocyte subpopulations and to better explain the different amounts of markers in these cells, we analyzed the cell cycle. KSC5ʹ cells display a higher percentage of cells in the G0-G1phase, as compared to ETA cells and KSC20ʹ, indicating a more quiescent state. Moreover, KSC5ʹ have the lowest percentage of cells in sub-G1, confirming that they are protected from cell death^27^ (Fig. 2B). The quiescent state of freshly isolated KSC5ʹ is further confirmed by the absence of Ki67, as opposed to the marked expression in ETA cells and in the other subpopulations (Fig. 2C). After 3 days in cultures, all subpopulations upregulate Ki67 is up-regulated in all keratinocyte subpopulations, due to higher proliferative rate (Supplementary Fig. S1C). Furthermore, K15, which identifies progenitor basal cells, is highly expressed in KSC 5ʹ, while it declines in ETA to further decrease in LTA and disappears in post-mitotic (PM) cells. K10 is nearly absent in KSC, slightly increases in ETA, while is abundantly expressed in LTA and PM cells. Involucrin, a late differentiation marker, is greatly expressed in LTA and PM cells, while it is significantly lower in ETA cells (Fig. 2D, 2E). These results show that KSC 5ʹ (from now on indicated as only KSC) are in a quiescent state and transit to a proliferating early progeny before committing to final differentiation.

### ETA Cells Display Unique Proliferative and Regenerative Features

**Figure 3.**
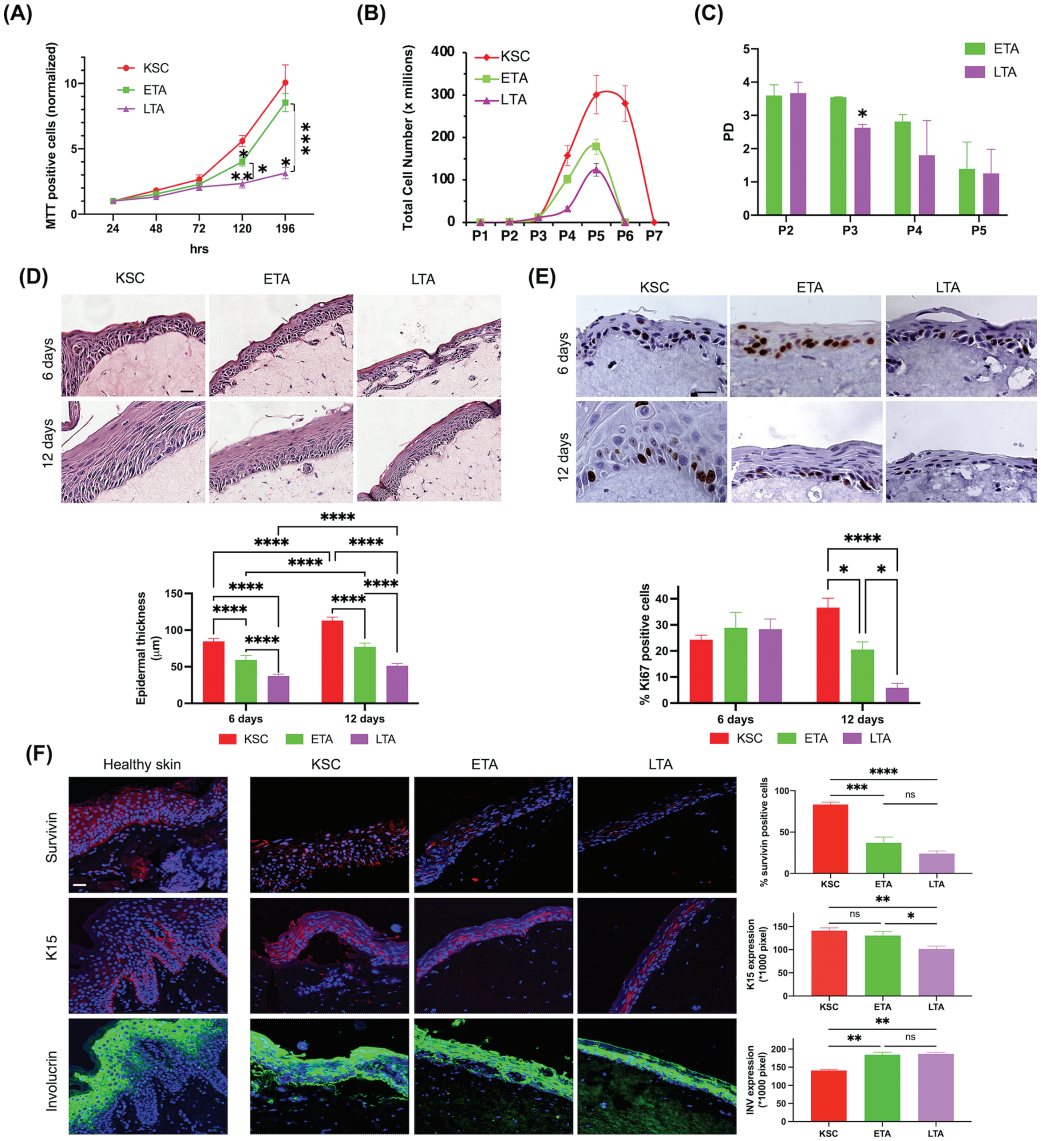
Functional behavior of ETA cells in 2D and 3D cultures. (**A**) Freshly isolated subpopulations were cultured up to 196 h after seeding and MTT assay was performed at different times. (**B**) Long-term assay was performed on freshly isolated keratinocyte subpopulations, as described in Materials & Methods. (**C**) The extent of proliferation of ETA and LTA cells left in a culture was estimated by calculation of the population doubling. (**D**) Reconstructed skin equivalent (SE) models generated from freshly isolated KSC, ETA, and LTA cells. After 6 or 12 days from keratinocyte seeding, skin equivalents were paraffin-embedded and sections were stained with H&E. Bar = 100 μm. Epidermal thickness was measured by D-Sight slide scanner software. (**E**) Immunohistochemical staining of Ki67 was evaluated as the percentage of positive cells, as described in Materials & Methods. Bar = 100 μm. (**F**) Sections of healthy skin and skin equivalents obtained from KSC, ETA, and LTA cells, were stained with anti-Survivin, anti-K15, and anti-Involucrin antibodies by IF. Bar = 100 μm. Stained areas were evaluated by image pixel count using Fiji-ImageJ. Data are represented as mean ± SEM. Ordinary one-way ANOVA followed by Tukey’s multiple comparisons are represented. ns: *P* > .05; * .01 < *P* < .05; ** *P* < .01; *** *P* < .001; ****P < .0001.

β1-integrin stimulates keratinocyte proliferation in healthy and diseased skin.^28-30^ β1-integrin is also associated with long-term growth potential in human epidermis.^31,32^We analyzed cell proliferation in keratinocyte subpopulations separated using the integrin-based adhesion capacity, as described. The number of viable cells reflecting proliferation by MTT assay is lower in ETA cells than in KSC. On the other hand, ETA cells display a higher proliferation rate than LTA cells (Fig. 3A). Consistently, long-term growth is the highest in KSC and statistically declines in ETA cells that, in turn, grow to a greater rate as compared to LTA cells (Fig. 3B). The estimation of population doubling (PD) levels shows similar ranges for ETA and LTA cells, except for passage 3 (P3), where ETA cells display a significantly higher PD rate than LTA cells (Fig 3C). This demonstrates that an early KSC daughter exists with intermediate growth potential.

We have previously shown that TA cells are capable of generating three-dimensional (3D) skin equivalents (SE).^18^ To better characterize the function of ETA cells, we created 3D skin reconstructs from KSC, ETA, and LTA cells. Six days after seeding, epidermal thickness in SE derived from ETA cells tends to be reduced, as compared to SE generated from KSC. However, the epidermis in SE derived from ETA is thicker than in LTA SE. At 12 days after seeding, the difference becomes more relevant (Fig. 3D). At 6 days after seeding, cells are still in their replicative status, as confirmed by the same rate of Ki67 positive cells, while Ki67 positive cells decrease at the later timepoints in ETA and LTA SE. However, ETA cells preserve a statistically significant higher proliferative status compared to LTA cells also at 12 days after seeding in the SE (Fig. 3E). Proliferation and differentiation markers were used to better characterize SE from the 3 keratinocyte subpopulations, using healthy human skin as a control. Survivin, which was originally shown to identify KSC,^25^ is more expressed in SE derived from these cells than in SE from ETA. ETA SE expresses a higher number of Survivin positive cells than LTA SE. Similarly, K15 expression is comparable among KSC and ETA SE and greater in ETA SE than in LTA SE. On the other hand, Involucrin is more expressed in LTA and ETA SE than in SE derived from KSC (Fig. 3F). Taken together, these results show that ETA cells contribute to epidermal regeneration to a better extent than LTA cells.

### Silencing of CD271 in ETA Cells Generates a More Proliferative SE

**Figure 4.**
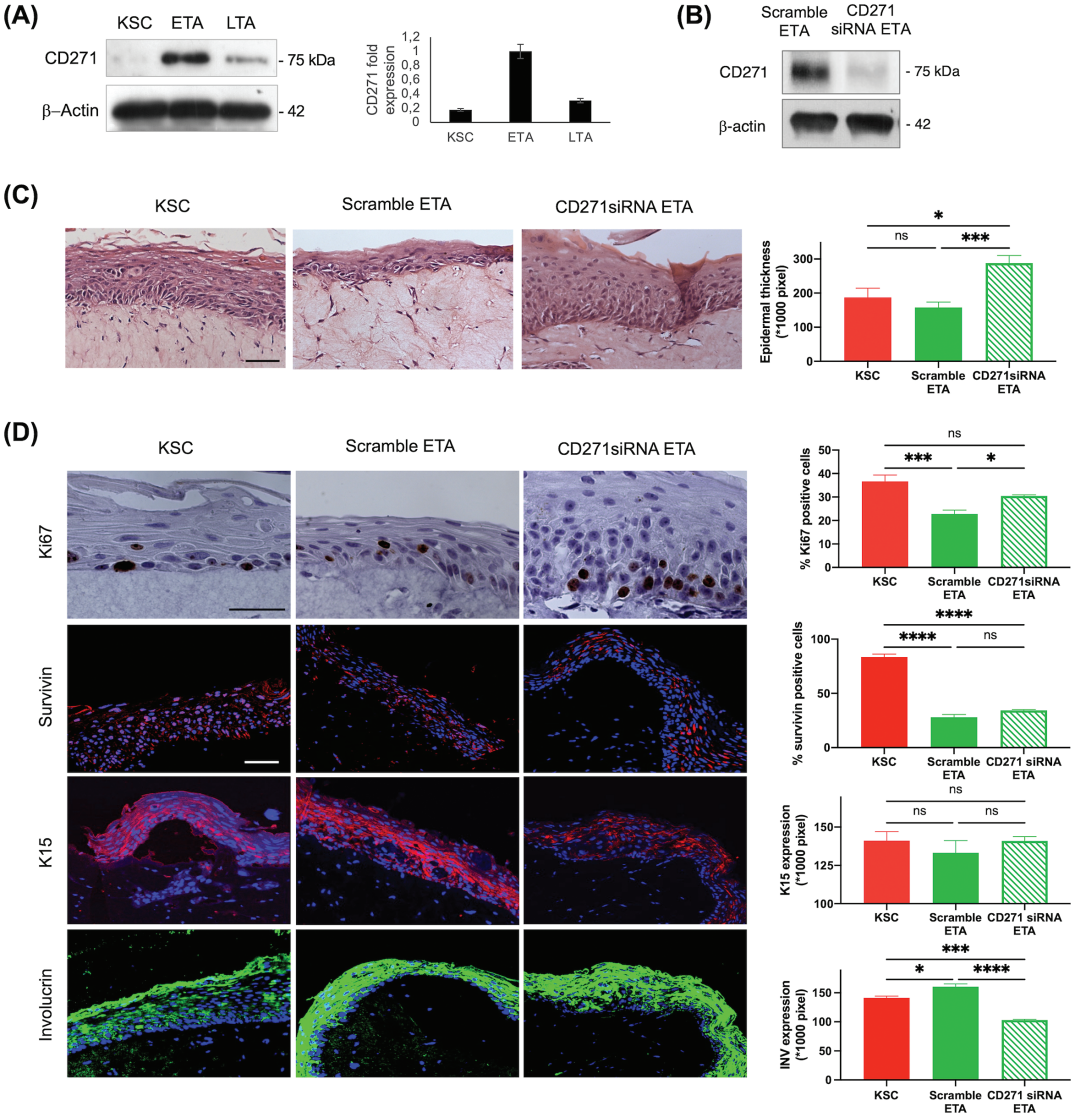
Generation of skin equivalents from CD271 silenced ETA cells. (**A**) Protein extracts from freshly isolated KSC, ETA, and LTA cells were immunoblotted for CD271 and the relative expression was densitometrically evaluated by Fiji-ImageJ software. (**B**) Confirmation of CD271 downregulation in protein extracts from scramble or CD271 ETA cells by immunoblotting. (**C**) Skin equivalents derived from KSC, CD271 silenced ETA, and scrambled ETA cells were paraffin-embedded at 6 and 12 days from cell seeding and sections were stained with H&E. Bar = 100 μm. Epidermal thickness was measured by Fiji-ImageJ software. (**D**) Sections of skin equivalents obtained from KSC, CD271 silenced ETA, and scrambled ETA cells were stained with anti-Ki67 antibodies by IHC, anti-Survivin, anti-K15, and anti-Involucrin antibodies by IF. Bar = 100 μm. The number of positive cells or stained areas was evaluated by image pixel count using Fiji-ImageJ. Data are represented as mean ± SEM. Ordinary one-way ANOVA followed by Tukey’s multiple comparisons test were performed, and comparisons are indicated. ns: *P* > .05; * .01 < *P* < .05; ** *P* < .01; *** *P* < .001; ****P < .0001.

CD271 is randomly expressed in the basal layer of the human epidermis, while no staining is detected in the suprabasal layers. More specifically, CD271 is predominantly expressed in TA cells, both at the mRNA and protein level with little expression in KSC.^17^ Moreover, CD271 acts as a mediator of KSC to early progeny transition.^18^ We reasoned that CD271 could identify the early progenitor cell (ETA) described here and might play an important role in the immediate differentiation process. For this purpose, we first evaluated the expression of CD271 in the three keratinocyte subpopulations, separated as described. CD271 protein levels are markedly expressed in ETA, while they are nearly undetectable in KSC and LTA cells (Fig. 4A). To better understand the role of CD271 in ETA, we silenced the receptor in these cells which were in turn used to generate SEs (Fig. 4B). The skin reconstructs were then compared to SE derived from KSC and from scrambled ETA cells (Fig. 4C). Epidermis is statistically thicker in CD271siRNA SE than in scramble ETA SE (Fig. 4D). Ki67 positive cells are more numerous in CD271siRNA SE than in scramble ETA SE. Moreover, Survivin is significantly less expressed in both ETA SE and in CD271siRNA SE than in KSC SE. Survivin positive nuclei, which have been shown to be more expressed in cultured proliferating KSC,^25^ statistically decline in an either scramble or CD271siRNA ETA SE. Similarly, K15 staining intensity is higher in CD271siRNA ETA SE than in scramble ETA SE. On the other hand, Involucrin is significantly more expressed in scramble ETA SE than in CD271siRNA ETA SE (Fig. 4D). These results indicate that CD271 identifies ETA cells and is important for keratinocyte homeostasis and epidermal differentiation.

### Overexpression of CD271 in LTA Cells Generates a Less Differentiated SE

**Figure 5.**
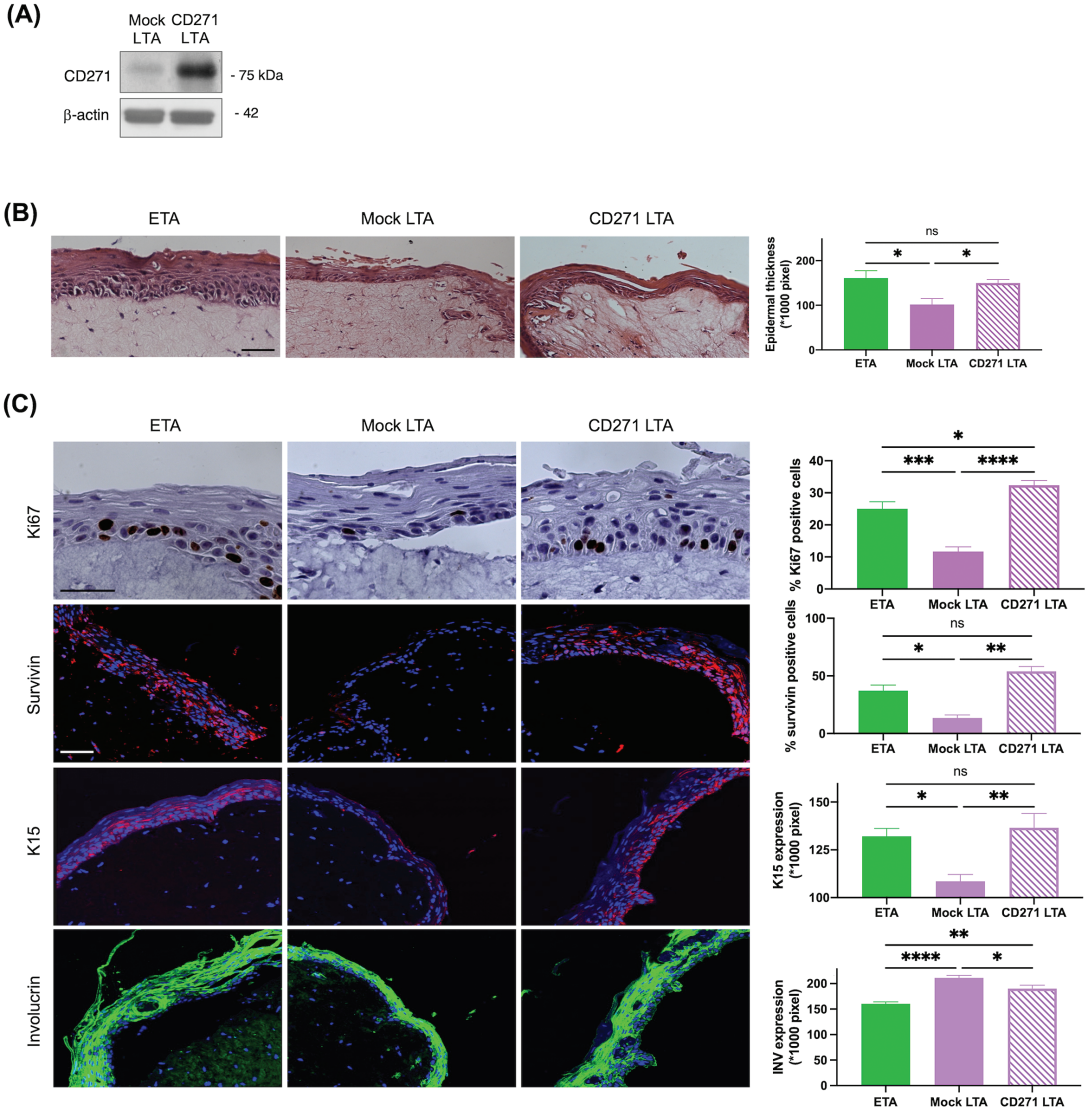
Generation of skin equivalents from LTA cells overexpressing CD271. (**A**) Confirmation of CD271 overexpression in protein extracts from mock or CD271 infected LTA cells by immunoblotting. (**B**) Skin equivalents derived from ETA, CD271 overexpressing LTA, and mock LTA cells were paraffin-embedded at 12 days from cell seeding, and sections were stained with H&E (B). Bar = 100 μm. Epidermal thickness was measured by Fiji-ImageJ software. (**C**) Sections of skin equivalents obtained from ETA, CD271 overexpressing LTA, and mock LTA cells, were stained with anti- Ki67 antibodies by IHC, anti-Survivin, anti-K15, and anti-Involucrin antibodies by IF. Bar = 100 μm. The number of positive cells or stained areas was evaluated by image pixel count using Fiji-ImageJ. Data are represented as mean ± SEM. Ordinary one-way ANOVA followed by Tukey’s multiple comparisons test were performed, and comparisons are indicated. ns: *P* > .05; *.01 < *P* < .05; ** *P* < .01; ***P < .001; ****P < .0001.

We have previously shown that CD271 positive TA cells are early differentiated keratinocytes that display a high proliferative potential.^18^ To further clarify the role of CD271 in keratinocytes, we retrovirally infected freshly isolated LTA cells to induce an upregulation of CD271 (Fig. 5A) and then we generated skin reconstructs from infected LTA cells, from mock LTA and from ETA cells (Fig. 5B). Epidermal thickness is superior in SE created from CD271 overexpressing LTA cells than in reconstructs from mock LTA and similar to that observed in SE from ETA cells (Fig. 5B). Ki67 staining shows that positive cells are more abundant in SE from CD271 infected LTA than in mock LTA SE. Survivin expression is significantly higher in SE from CD271 infected LTA cells than in the mock counterpart. Similarly, K15 expression is significantly higher in SE from infected LTA than in mock LTA SE. Finally, the staining intensity of Involucrin is stronger in SE from mock LTA than in SE from CD271 overexpressing LTA (Fig. 5C). These results suggest that CD271 can revert LTA cells to a less differentiated and more proliferative phenotype.

### CD271 Decreases with Cellular Senescence

**Figure 6.**
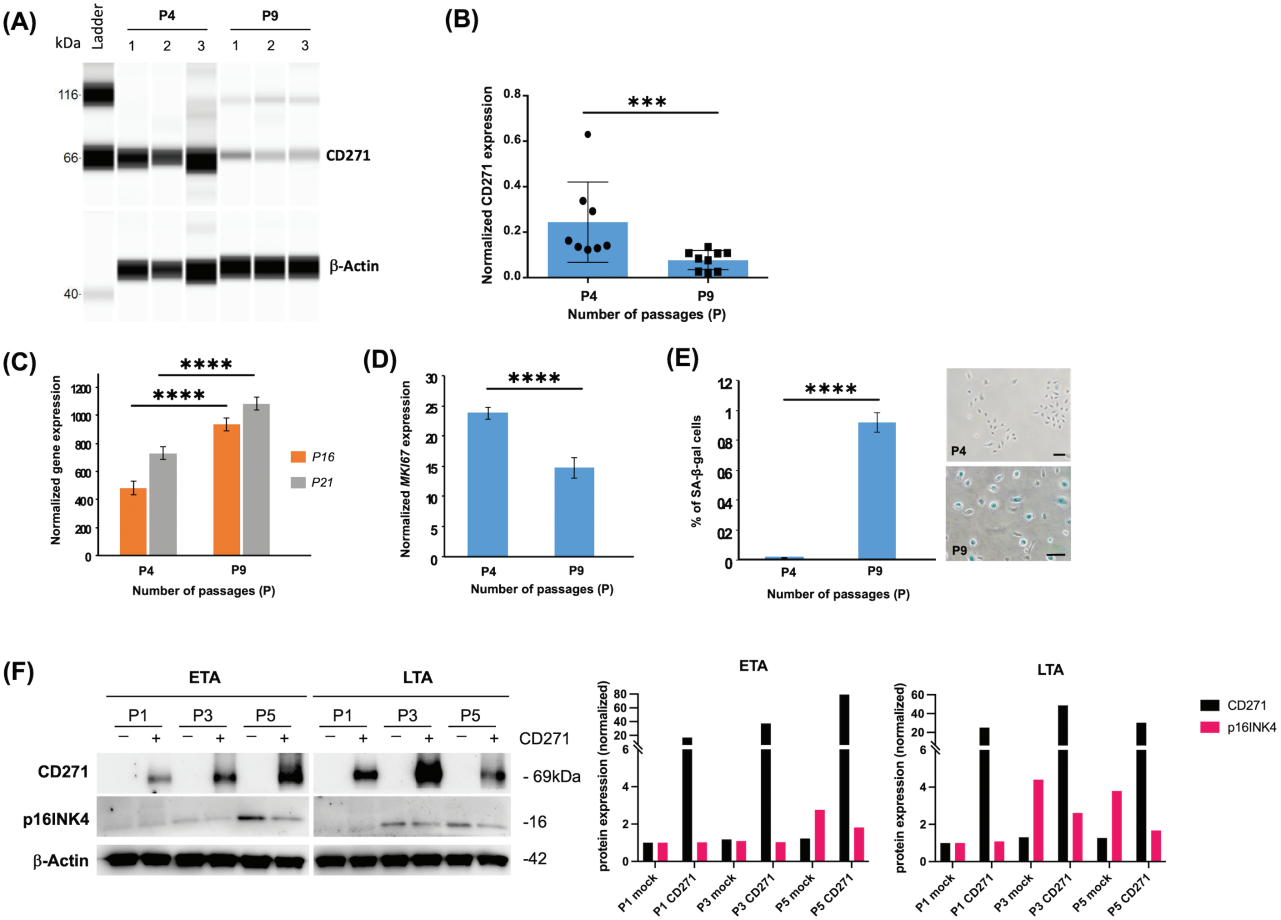
CD271 expression in replicative senescence. (**A**) CD271 expression signal strength in normal (*n* = 4 passages or P4) and in replicative-induced senescent (*n* = 9 passages or P9) human epidermal keratinocytes (HEK) was analyzed by capillary Western blotting. (**B**) Results are presented as means and SDs of independent samples of triplicate cell cultures in three experiments. CD271 signals were normalized using the β-actin signals. *P* = .0003). (**C**) (**D**) P16, P21, and Ki67 gene expression were analyzed in normal (*n* = 4 passages or P4) and replicative-induced senescent (*n* = 9 passages or P9) human epidermal keratinocytes (HEK). Gene expression was quantified by RT-qPCR and normalized with β2M expression. (**E**) Microscopical analysis of NHEK stained with the chromogenic substrate (X-Gal) for senescence-associated β-galactosidase (SA-β-Gal) activity determination and quantification as % of SA-β-Gal positive cells by image analysis, representative pictures are reported on the right. Bar = 100 μm. Statistical significance was calculated using Student’s *t*-test (*P* < .0001). (F) CD271 overexpression in protein extracts from mock (−) or CD271 infected (+) ETA and LTA cells at different passages (P1, P3, and P5), was analyzed by immunoblotting. In parallel, analysis of p16INK4 expression in the same samples. Densitometric analysis is shown. Data were normalized on mock P1 expression levels per protein.

A number of genes are implicated in the senescence and aging of human skin.^33^ Little is known however on CD271 expression during keratinocyte senescence. We have measured CD271 protein levels in keratinocytes at progressive passages (Fig. 6A). CD271 protein is strongly expressed in normal (passage 4, P4) keratinocytes, while it dramatically decreases in replicative-induced senescent keratinocytes at passage 9 (Fig. 6A, 6B). Replicative-induced senescence in human keratinocytes from passage 4 to 9 is confirmed by a significant increase of the gene, a cell cycle checkpoint and a reliable marker of cellular aging,^26^ and of p21, a cyclin-dependent kinase inhibitor that acts as an effector of chronic senescence^34^ (Fig. 6C). By contrast, Ki67 gene expression tends to significantly decrease during the same replication passages (Fig. 6D). Furthermore, senescence-associated β-galactosidase (SA-B-Gal) activity in human keratinocytes is not detectable at passage 4, while it becomes significantly elevated at passage 9 (Fig. 6E). To further evaluate the role of CD271 during replicative senescence, we overexpressed CD271 during progressive passages in ETA and LTA cells. Western blot analysis indicates an inverse correlation between CD271 and p16INK4 which is particularly evident at passage 5 (P5) in ETA cells, while it is observed already at passage 3 in LTA cells. (Fig. 6F) These results suggest that reduced CD271 is a prerequisite for senescence in human keratinocytes.

## Discussion

Interfollicular epidermis (IFE) is renewed by KSC and their progenitors in the basal layer within a local microenvironment known as a niche. Niche components regulate the fate of KSC and their commitment to differentiation. Because KSC play a critical role in the maintenance and regeneration of the epidermis, it is of paramount importance to dissect the events associated with their early differentiation. Investigations in the field have mostly focused on hair follicle (HF) stem cells and/or in the mouse model.^35^ Although IFE KSC are expected to exert a more important task in skin homeostasis, little is known about the initial events associated with KSC exiting the niche, particularly in human skin. In the present study, we describe an early progenitor (ETA cells) isolated by its integrin levels and adhesion to type IV collagen with unique proliferative potential and capable of regenerating the human epidermis. While previous studies have shown the enrichment for β1-integrin in KSC after 20-min adhesion to type IV collagen,^22,36^ we demonstrate that this subpopulation comprises both KSC5ʹ and an early TA subpopulation (ETA), while keratinocytes adhering in 5 min are the actual KSC (KSC5ʹ). Consequently, TA cells isolated after overnight adhesion in the original studies,^37^ display the features of more differentiated keratinocytes (LTA). Other markers have been used to distinguish KSC from their progenitors,^38-40^ although the isolation of TA cells remains elusive. FOXM1 has been recently shown to be an exclusive marker of holoclones by transcriptomic analysis.^23^ On the other hand, in the present work, we demonstrate that this transcription factor is expressed in ETA, but not in KSC cells. The apparent discrepancy is well explained by the different approach: indeed, our characterization is performed on enriched stem keratinocyte population directly isolated from skin biopsies and immediately analyzed, thus on quiescent cells, while holoclones are clonogenic cultured keratinocytes expanded in vitro. This is further supported by the prevalent expression of ΔNp63 and Survivin in holoclones and in ETA cells, which are both in a proliferative state. Using single-cell-RNA sequencing, Wang and co-workers have recently identified four stem cell populations in basal keratinocytes freshly isolated from the human neonatal foreskin, proposing a hierarchic model of stem cells with different proliferation capacities.^41^ Although our study does not allow to either support or oppose the presence of multiple stem cell pools, the transition of KSC to ETA cells seems to be consistent with the gradual process of differentiation proposed by Wang and co-workers. Interestingly, given the same method of keratinocyte isolation, the label-retaining stem cells with low proliferative capacity, as identified by Wang et al., are enriched in ITGB1 (β_1_-integrin), alike KSC presented here.

ETA cells described here appear to display a similar behavior to keratinocytes expressing high levels of phosphorylated p63 (pp63). Indeed, Suzuki and Senoo have shown that human KSC initial differentiation is accompanied by pp63, rapid decrease of β1-integrin expression, and reduced proliferative potential.^42^In the mouse system, 2 distinct interfollicular KSC populations with different rates of proliferation, differentiation, and upward transport have been described.^43^We might infer that the short-lived early progenitors expressing medium integrin levels characterized by Sada and co-workers correspond to the ETA cells presented in the current paper. It should be noted that two TA subpopulations with different features exist in HFKSC^44^ and in epithelial tissue other than the epidermis.^45^

A complex NT network has been shown to perform diverse functions in human skin.^46^ Keratinocytes release NT that, in turn, exert autocrine activities through the high-affinity receptors Trks and CD271.^47^ We have previously shown that KSC express the highest levels of nerve growth factor^48^ that acts as a mitogen and a survival factor for the neighbor cells.^49^ We have also provided evidence that CD271 mediates apoptosis in human keratinocytes.^17^ This suggests that an NT network can definitely take part in the KSC and progenitor niche and regulate epidermal homeostasis.^50^ In particular, we propose that CD271 expressed in ETA cells is a critical mediator of the niche. Indeed, CD271 overexpression induces the switch of KSC to TA cells, and CD271 silencing reverts TA cells to a KSC phenotype.^18^ Moreover, CD271 reverts LTA cell-generated SE into a less differentiated phenotype, while silencing of CD271 renders ETA cell-generated SE more similar to KSC skin reconstructs. This feedback loop among KSC, ETA, and LTA cells reminds us of the interdependency concept proposed by Hsu and co-workers, where TA cells expressing Sonic Hedgehog are critical components of the niche and regulate KSC fate.^51^ In agreement with this concept, we envisage that ETA cells expressing CD271 serve as an active signal for KSC differentiation and epidermal regeneration.

CD271 also appears to rescue ETA and LTA keratinocytes from senescence, thus contributing to the maintenance of epidermal homeostasis. While the precise mechanism of action remains to be elucidated, the anti-oxidant selenium was shown to protect keratinocyte senescence through cell adhesion mediated by β1-integrin binding to type IV collagen and by reducing p16INK4 gene.^52^ Moreover, in human keratinocytes, CD271 down-modulates NF-kB^17^ which is normally correlated with cell senescence through the activation of senescence-associated secretory phenotype (SASP).^53^

Psoriasis is a T helper type17-mediated inflammatory disease, keratinocytes being essential and possibly early players in the pathogenesis.^54^ β1-integrin is aberrantly expressed in the suprabasal epidermal layers in psoriasis, while ectopic expression of β1-integrin recapitulates a psoriasiform phenotype in different animal models,^55,29^indicating that integrin signaling plays a key role in psoriasis. Little is known about the correlation between integrin and CD271.^56^ We recently showed that psoriatic TA cells lack CD271 and are resistant to apoptosis, while re-expression of CD271 renders these cells susceptible to cell death.^17^ Moreover, SE derived from CD271 depleted TA cells display a psoriasiform phenotype.^18^ Consistently, the present study demonstrates that in SE generated from ETA cells silenced for CD271, the epidermis is thicker and proliferative markers are highly expressed, as compared to mock SE from ETA. Given that expansion of the TA cell compartment is a distinct feature of psoriasis,^57^ this should open the way to further investigation on the alteration of the keratinocyte subpopulations in psoriasis.

In conclusion, we have identified and characterized an early TA cell as the initial KSC daughter exiting the niche and starting the differentiation process. We also confirm that CD271 plays a critical role in the niche and, because of its critical functions in keratinocyte differentiation, apoptosis, and senescence, regulates epidermal homeostasis.

## Supplementary Material

sxac060_suppl_Supplementary_Material

## Data Availability

The data that support the findings of this study are available on request from the corresponding author.
