# Supplementary material for: Isolation of an “Early” Transit Amplifying Keratinocyte Population in Human Epidermis: A Role for the Low Affinity Neurotrophin Receptor CD271
Source: Stem Cells. 2022 Aug 29;40(12):1149–61. doi: 10.1093/stmcls/sxac060 (PMC9806768; doi:10.1093/stmcls/sxac060)
Supplement: sxac060_suppl_Supplementary_Material [file sxac060_suppl_supplementary_material.pdf]

SUPPLEMENTARY MATERIAL

Supplementary Figure 1

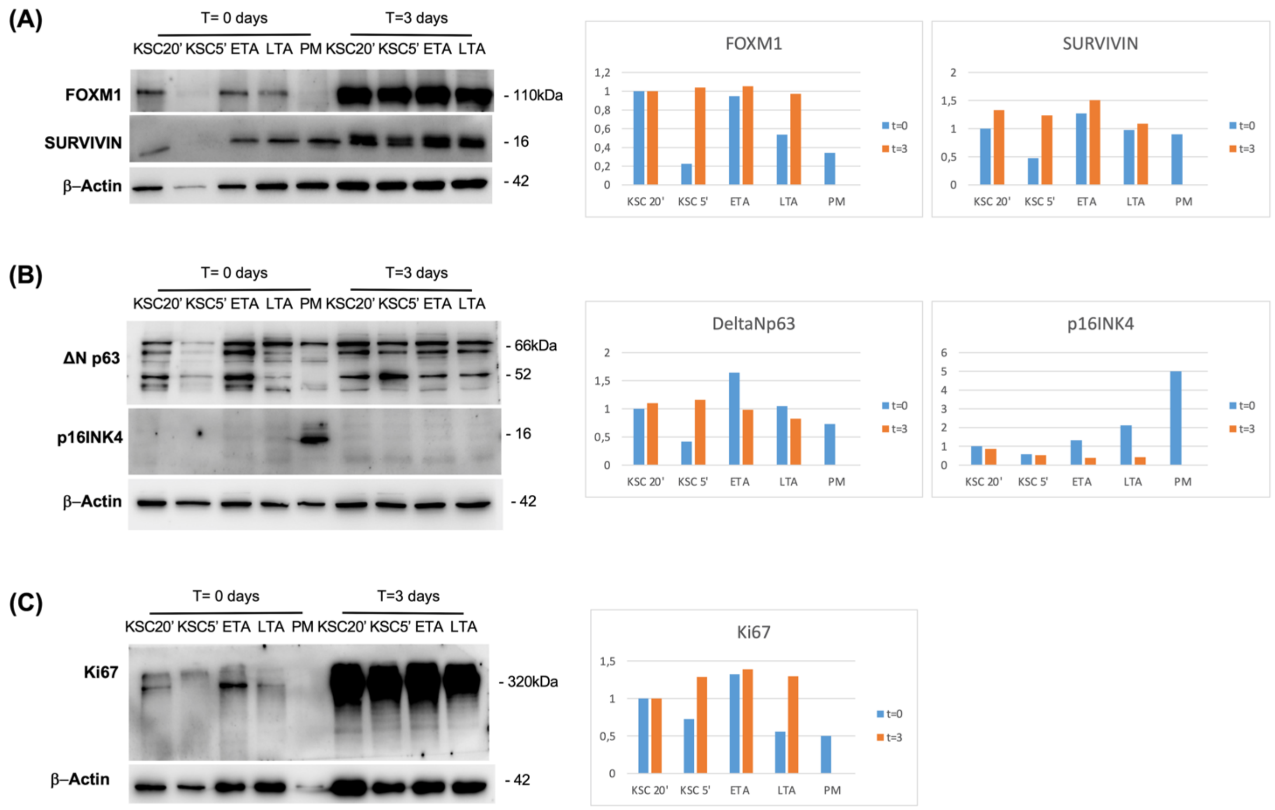

Supplementary Figure 1. Molecular characterization of freshly isolated keratinocyte subpopulations and after 3 days of culture. Keratinocytes were isolated based on their ability to adhere to type IV collagen. Subpopulations were obtained: KSC20’, KSC5’, ETA, LTA, PM. Cells were lysed immediately after the subpopulation isolation (T=0 days) and after 3 days of culture (T=3 days). The expression of FOXM1, Survivin (A), deltaNp63 (ΔNp63), p16INK4 (B) and Ki67 (C) were analyzed by WB. Actin was used as internal control. Densitometric analysis per each protein was shown, evaluated by Fiji-ImageJ software.
